# Phospho-enol pyruvate carboxykinase inhibition limits effector function in inflammatory T cells

**DOI:** 10.3389/fimmu.2026.1706167

**Published:** 2026-04-13

**Authors:** Rebecca J. Brownlie, Helen Carrasco Hope, David Wright, Graham P. Cook, José C. Perales, Robert J. Salmond

**Affiliations:** 1 Leeds Institute of Medical Research at St. James’s, University of Leeds, St. James’s University Hospital, Leeds, United Kingdom; 2 Faculty of Science and Medicine, Université de Fribourg, Fribourg, Switzerland; 3 Institute of Immunology and Infection Research, University of Edinburgh, Ashworth Laboratories, Edinburgh, United Kingdom; 4 Department of Physiological Sciences, School of Medicine, University of Barcelona, L’Hospitalet del Llobregat, Spain

**Keywords:** immunometabolism, PEPCK, phospho-enol pyruvate carboxykinases, T cell, T cell activation

## Abstract

**Introduction:**

Following antigenic stimulation, T cells switch from a catabolic metabolic state maintained by low levels of nutrient uptake to an anabolic metabolism that sustains the biosynthetic and energetic demands of clonal expansion, differentiation, and effector function. Much progress has been made in understanding the transcriptional and enzymatic regulation of activated T cell metabolism. However, less is understood of the role for regulators of anaplerosis and cataplerosis such as phospho-enol pyruvate carboxykinases (PEPCK) in T cells.

**Methods:**

Assessment of PEPCK expression in mouse T cells was performed. Pharmacological inhibitors were used to assess functional and metabolic roles for PEPCKs in T cell activation.

**Results:**

We show that mitochondrial PEPCK (PEPCK-M) is upregulated following T cell activation, while cytosolic PEPCK-C was not detected. The PEPCK inhibitors limited CD8^+^ T cell cytotoxic capacity and both CD4^+^ and CD8^+^ T cell inflammatory cytokine production. The suppression of T cell effector functions by PEPCK inhibitors was associated with decreased maximal mitochondrial respiration.

**Discussion:**

These data suggest that PEPCKs act to modulate mitochondrial metabolism, supporting effector function in T cells.

## Introduction

The processes of T cell activation and differentiation are linked to the regulation of cellular metabolism. These pathways provide the energy required for growth, proliferation, and effector functions, while metabolites can also directly modulate T cell differentiation. Importantly, the dysregulation of cellular metabolism has been linked to immune decline in aging (1, 2), the failure of anti-tumor T cell responses (3, 4), and autoimmunity (5, 6).

The changes in T cell functional state that follow T cell antigen receptor (TCR) triggering are accompanied by metabolic reprogramming. TCR and costimulatory CD28 signals promote the activation of mTOR and Myc signaling pathways that result in upregulation of nutrient receptors and activation of glycolytic and glutaminolysis pathways (7, 8). T cell metabolic reprogramming is also influenced by cytokines such as transforming growth factor β (9, 10). Both CD4^+^ T helper (Th) cells and CD8^+^ cytotoxic T lymphocytes (CTLs) rely upon aerobic glycolysis to meet the metabolic demands of proliferation and effector differentiation, while mitochondrial biogenesis and metabolism are also required for T cell activation (11–14). The tricarboxylic acid (TCA) cycle serves to link the catabolism of carbohydrates, fats, and proteins to oxidative phosphorylation (OXPHOS) via the production of NADH and FADH. Glycolysis and the TCA cycle also provide precursors for biosynthetic pathways. The removal of intermediate metabolites in metabolic pathways is termed cataplerosis, while effector T cells utilize these pathways concurrent with those that replenish these metabolites (anaplerosis)—for example, T cell growth and proliferation are dependent upon the anaplerotic production of the TCA cycle intermediate α-ketoglutarate from glutamine in glutaminolysis (15, 16).

A wealth of data has defined the role of transcriptional and enzymatic regulators of glycolysis and glutaminolysis in T cell activation. By contrast, the specific roles of regulators of anaplerosis and cataplerosis in T cells are poorly understood. Two isoforms of phosphoenolpyruvate carboxykinase (PEPCK) are central players in the regulation of this axis as enzymes that catalyze the conversion of oxaloacetate (OAA) to phosphoenolpyruvate (PEP) (17). Cytoplasmic PEPCK (PEPCK-C; encoded by *PCK1*/*Pck1*) is a critical enzyme in gluconeogenesis, while mitochondrial PEPCK (PEPCK-M; encoded by *PCK2/Pck2*) has a regulatory function in mitochondrial metabolism. In tumor cells, PEPCK-C promotes glutaminolysis and the TCA cycle flux (18), while the downregulation of TCA cycle anaplerosis/cataplerosis and a subsequent reduction in OXPHOS were associated with decreased PEPCK-M expression in melanoma (19). It is worth noting that the ectopic overexpression of PEPCK-C can partially overcome the requirement for glycolysis in T cell activation. PEP produced during glycolysis or following enforced PEPCK-C expression facilitates prolonged TCR-induced Ca^2+^ signaling and nuclear factor of activated T cells (NFAT) activation (20). High levels of PEP, produced during glycolysis or following PEP supplementation, have been shown to impede Th17 cell differentiation, whereas a paucity of PEP, under conditions of glucose starvation, impairs Th1/CTL responses (20, 21), highlighting the key importance of regulating PEP levels in T cells. Furthermore, recent studies have shown that PEPCK-C expression is elevated in memory T cell populations where it functions to increase glycogen synthesis and metabolism to fuel the pentose phosphate pathway (22). PEPCK-C expression is important in memory T cells for the maintenance of high levels of glutathione (GSH) and redox homeostasis (22, 23). By contrast, less is known of the role of PEPCK isoforms in effector and inflammatory T cell activation and metabolism.

In the current work, we assessed the expression levels of PEPCK isoforms in mouse T cell subsets and used pharmacological inhibition to ascertain the importance of PEPCK function during T cell activation. Data indicate that PEPCK-M is expressed in effector T cells, while PEPCK inhibition limited CD8^+^ T cell cytotoxic capacity *in vitro* and both CD4^+^ and CD8^+^ T cell inflammatory cytokine production. The suppressive effects of PEPCK inhibitors on T cell function were distinct from previously described roles of PEPCK-C in maintaining GSH levels but were associated with decreased maximal mitochondrial respiration.

## Materials and methods

### Database searches

**Figure 1:**
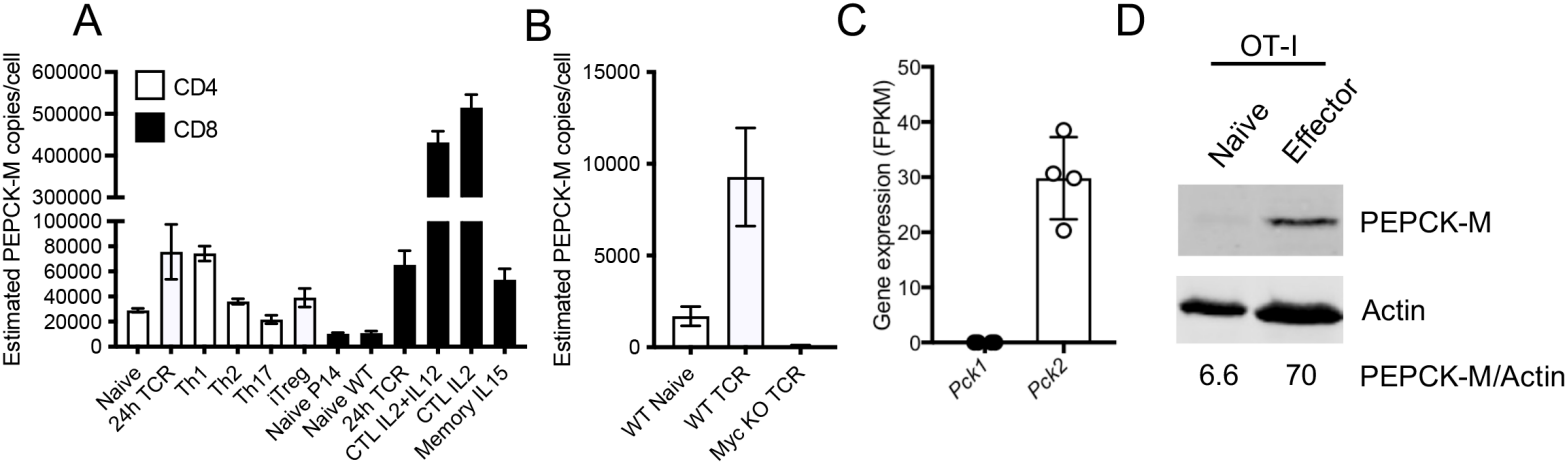
Expression of PEPCK-M in effector T cells. PEPCK-M protein copy numbers/cell in mouse T cell subsets **(A)** and in control and Myc-deficient mouse CD8^+^ T cells stimulated for 24 h with CD3 and CD28 antibodies **(B)**. The data are from Immunological Proteome Resource (immpres.co.uk), and the datasets used to generate **(B)** were published in (26). The values are means, and the error bars represent SD from *n* = 3 biological replicates (T cells from an individual mouse). **(C)** Expression of *Pck1* (encoding PEPCK-C) and *Pck2* (encoding PEPCK-M) transcripts expressed as fragments per kilobase million (FPKM) in OT-I T cells activated for 24 h with SIITFEKL antigen. The circles represent individual values from four biological replicate samples. Data are from RNA-sequencing datasets originally published in (9). **(D)** Effector OT-I CTLs were generated by 2 days of OVA-peptide stimulation, followed by 4 days of expansion and differentiation in IL-2. Western blot analysis of PEPCK-M expression in control naïve and the resultant effector CTLs was performed. β-actin reprobes serve as protein loading controls. PEPCK-M expression represent densitometric values of PEPCK-M/actin. The data are from one of three repeated experiments.

Quantitative proteomic data were taken from the Immunological Proteome Resource (https://immpres.co.uk) (24). The data shown are protein copy number/cell expressed by mouse T cell populations within the hematopoietic cell proteomes dataset, using “Pck1” and “Pck2” as search terms, for PEPCK-C and PEPCK-M, respectively. The protein copy numbers are estimated using the “proteomic ruler” method (25). The data in **Figure 1B** are PEPCK-M protein copy numbers within the Myc-regulated T cell proteome dataset, originally published in (26) and available within ImmPRes. The data shown in **Figure 1C** are taken from RNA-Seq datasets originally published by Hope et al. (9) and represent fragments per kilobase million values for *Pck1* (encoding PEPCK-C) and *Pck2* (encoding PEPCK-M) transcripts within OT-I T cells stimulated for 24 h with SIITFEKL peptide.

### Mice

OT-I *Rag1*^-/-^ and C57BL/6J mice were maintained at the University of Leeds’ St. James’s Biomedical Services animal facility. All experiments were performed using both male and female mice at 7–15 weeks of age.

### Cell lines

ID8-OVA-fLuc cells (27) were maintained in Iscove’s modified Dulbecco’s medium (IMDM, Gibco) supplemented with 5% fetal calf serum (FCS) (Gibco), L-glutamine, and antibiotics (100 U penicillin, 100 μg/mL streptomycin). EL4-OVA cells, originally from Klaus Okkenhaug (University of Cambridge), were maintained as detailed above, with the addition of 400 μg/mL G418 antibiotic. The cell lines were negative for mouse pathogens, including mycoplasma contamination.

### T cell culture and stimulation

OT-I T cells were obtained from the lymph nodes of OT-I *Rag1*^-/-^ mice. Single-cell suspensions were prepared by dissociating lymph nodes using a 70-μM filter, with the cell purity of CD8^+^ T cells being >90%. The OT-I T cells were cultured in IMDM supplemented with 5% FCS, L-glutamine, antibiotics (100 U penicillin, 100 μg/mL streptomycin) and 50 μM 2-mercaptoethanol. The OT-I T cells were activated using SIINFEKL peptide (Cambridge Peptides) for the time periods indicated in the figure legends. In some experiments, the control OT-I T cells were left unstimulated *in vitro*. 3-Mercaptopicolinic acid (Cayman Chemicals) was added to culture media at a final concentration of 100 μM unless otherwise stated. The PEPCK inhibitor 5-chloro-*N*-{4-[(cyclopropylmethyl)-1-(2-fluorbenyzl)-2,6-dioxo-2,3,6,9-tetrahydro-1*H*-purin=8=yl)methyl]phenyl}-1,3-dimethyl-1*H*-pyrazole-4-sulfonamide (iPCK2) was synthesized as previously described (28) and was added to the culture media at a final concentration of 5 μM. Where indicated, 1 μM L-glutathione (GSH, Tocris), 50 μM glycogen phosphorylase inhibitor (Cayman Chemicals), and 1 mM phospho-enol pyruvate monopotassium salt (PEP, Merck) were added to culture. For the generation of cytotoxic T lymphocytes (CTLs), OT-I T cells were activated ± 3-MP or iPCK2 with 10–^8^ M SIINFEKL for 2 days, followed by 4 days of differentiation with 20 ng/mL recombinant human IL-2. For the analysis of cytokine production, day 6 CTLs were re-stimulated with 10^-7^–10–^8^ M SIINFEKL for 4 h in the presence of 2.5 μg/mL brefeldin A (Sigma). For Th1 and Th17 differentiation, CD4^+^ T cells were purified from C57BL/6 lymph nodes by negative selection using magnetic beads (Miltenyi Biotech). T cells were activated in 48-well plates for 3 days (Th1) or 6 days (Th17) with plate-bound CD3ε (Clone 145-2C11, BioLegend) and CD28 mAb (Clone 37.51, BioLegend) with the following recombinant cytokines (all Peprotech): Th1—10 ng/mL mouse IL-12, 10 ng/mL human IL-2; Th17—1 ng/mL mouse TGFβ, 20 ng/mL mouse IL-6, 5 μg/mL anti-IFNγ (Clone XMG1.2, BioLegend), 5 μg/mL blocking anti-CD25 (Clone PC61, BioLegend). Brefeldin A was added to the Th1 and Th17 cultures for the final 4 h of culture prior to intracellular staining and flow cytometry.

### Western blotting

Lysates from naïve OT-I T cells or from day 6 activated OT-I T cells, generated as described above, were prepared in RIPA lysis buffer, and protein concentrations were assessed by using Bradford Assay (Thermo Fisher) and 15 μg protein/sample loaded on polyacrylamide gels. Western blotting was performed as previously described using Li-COR Odyssey Imaging System (29). The antibodies used were rabbit polyclonal anti-PEPCK-M (#6924, Cell Signaling Technology), mouse anti-β-actin (Clone AC-15, Sigma), goat anti-rabbit AF680, and goat anti-mouse AF790 (Molecular Probes).

### Flow cytometry

The following antibodies were used: CD4-allophycocyanin (APC) (Clone GK1.5), CD8β-phycoerythrin (PE) cyanine 7 (Cy7) (Clone YTS156.7.7), CD69-peridinin chlorophyll protein (PerCP) Cy5.5 (Clone H1.2F3), CD71-fluorescein isothiocyanate (FITC) (Clone R17217), PD-1-PE (29F.1A12), granzyme B-Pacific Blue (Clone GB11), Tbet-PE (Clone 4B10), IFNγ-alexa flour 488 (AF488) (Clone XMG1.2), TNFα-PerCP Cy5.5 (Clone MP6-XT22), IL-17A-APC (Clone TC11-18H10.1) (all BioLegend), and retinoic acid-related orphan receptor (ROR) gamma-PE (Clone B20, eBioscience). For live cell discrimination, the cells were stained with live/dead aqua dyes (Life Technologies). For intracellular staining, the cells were fixed in eBioscience FoxP3 fix/permeabilization buffers prior to staining in the permeabilization buffer. The samples were acquired with LSRII (Becton Dickinson) or Cytoflex S (Beckman) flow cytometers, and data were analyzed using FlowJo Software (Treestar).

### IL-2 ELISA

OT-I T cells were activated with 10–^6^ M SIINFEKL ± 3-MP in the presence of 2.5 μg/mL CD25 blocking antibody to prevent IL-2 consumption, and supernatants were collected at 24 h. The levels of supernatant IL-2 were assessed by ELISA using the mouse IL-2 DuoSet kit according to the manufacturers’ instructions (R&D Systems).

### Quantitative RT-PCR

OT-I T cells were activated with 10–^6^ M SIINFEKL ± 3-MP for 24 h, and cell pellets were stored at -80°C. RNA was prepared using PureLink^®^ RNA Mini Kit (Invitrogen), and cDNA synthesis was performed using the RevertAid First Strand cDNA synthesis kit (Thermo Scientific). *Gzmb* expression was quantified using the ddCT method and Taqman reagents using the QuantStudio 7 Real-Time PCR system ((Applied Biosystems). The relative expression of *Rpl13a* was used to normalize the gene expression across samples. The following Taqman probes were used: *Gzmb* – Mm00442837_m1; *Rpl13a* – Mm05910660_g1.

### *In vitro* cytotoxicity assay

Target ID8-OVA-fLuc cells were seeded in 48-well plates for 6 h, prior to the addition of day 6 CTLs at the ratios indicated in the figures. Following overnight culture, the plates were gently washed in PBS to remove T cells and target cell debris prior to the assessment of luciferase activity by the addition of luciferin (Regis Technologies) and IVIS imaging. Specific cell lysis was calculated by the comparison of luminescence wells in experimental wells to target only and blank wells.

### EL4-OVA tumor model and ACT experiments

EL4-OVA cells (1 × 10^6^) were injected subcutaneously into the flank of C57BL/6 mice. After 5 days, the mice were randomly divided into three groups; the control mice received no ACT, while the additional groups received intravenous injections via the tail vein of either control or 3-MP CTLs (5 × 10^6^/mouse). Tumor mass was assessed by caliper measurements every 2 to 3 days, until the tumors in any control group mice reached a diameter of 15 mm.

### Seahorse metabolic assays

Mitostress test and glycolysis stress kits (Agilent) were used to measure metabolic profiles using a Seahorse XFe96 analyzer. The activated T cells were washed (3×) in PBS prior to transfer to the Seahorse assay plates (1 × 10^5^/well) and adhered using Cell-Tak solution (22.4 μg/mL, Corning) in complete XF assay medium. Oligomycin (1 μM), carbonyl cyanide-p-trifluoromethoxyphenylhydrazone FCCP (1.5 μM), and rotenone/antimycin A (500 nM) were injected using the Mitostress test protocol. For glycolysis stress tests, glucose was omitted from the base media. Glucose (10 mM), oligomycin (1 μM), and 2-deoxyglucose (50 mM) were injected using the glycolysis test protocol. Data were collected in Wave software and analyzed using GraphPad Prism.

### Data analysis and statistics

Statistical significance (*p*-value < 0.05) was determined by paired or unpaired Student’s *t*-test and one- or two-way ANOVA with Tukey’s multiple-comparisons tests using GraphPad Prism, as stated in the figure legends. The dots in graphs represent biological replicate samples, and the error bars represent SDs, unless otherwise stated. The values for biological replicates represent the mean values of technical replicates performed in a single independent experiment.

### Study approval

The mouse breeding and experiments performed were reviewed and approved by the University of Leeds Animal Welfare and Ethical Review Committee and were subject to the conditions of UK Home Office Project License PDAD2D507, held by RJS.

## Results

### Expression of PEPCK-M in effector T cells

We used publicly available datasets to assess the expression of cytosolic and mitochondrial PEPCK isoforms in mouse T cells. Quantitative mass spectrometry data from the Immunological Proteome Resource (ImmPRes) (24) showed that PEPCK-M was expressed at low levels in naïve CD4^+^ and CD8^+^ T cells (**Figure 1A**). The expression levels of PEPCK-M were elevated in most activated mouse T cell populations, compared with naïve cells, with the highest levels being in effector CD8^+^ cytotoxic T lymphocyte (CTL) populations. The analysis of a further ImmPRes proteomic dataset, originally published by Marchingo and colleagues (26), showed that the upregulation of PEPCK-M in CD8^+^ T cells following TCR stimulation was strictly Myc-dependent (**Figure 1B**). By contrast, PEPCK-C was not detected in any of the T cell mass spectrometry datasets available within ImmPRes. Consistent with the proteomic data, the analysis of our published RNA-sequencing data (9) demonstrated that the *Pck1* transcripts were absent whereas the *Pck2* transcripts were readily detectable in activated CD8^+^ OT-I TCR transgenic T cells (**Figure 1C**). Furthermore, Western blot analysis demonstrated that the PEPCK-M protein was expressed at low but detectable levels in naïve OT-I T cells and upregulated in CTLs (**Figure 1D**, **Supplementary Figure 1**). These data together suggest that PEPCK-M is expressed in effector T cells and that PEPCK-M/*Pck2* upregulation is part of the TCR-driven, Myc-dependent transcriptional program that regulates T cell metabolic reprogramming.

### PEPCK inhibitors limit CD8^+^ T cell cytolytic activity

**Figure 2:**
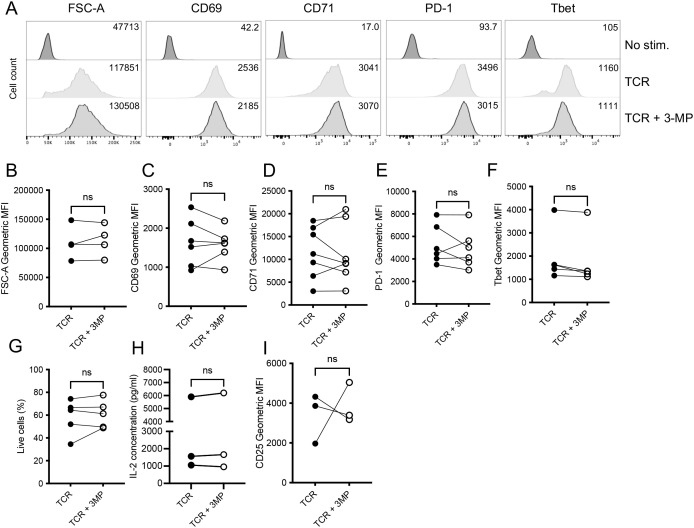
PEPCK inhibitors do not prevent T cell activation marker expression. The OT-I TCR transgenic T cells were stimulated with cognate peptide SIINFEKL (TCR) ± 3-MP (100 μM) for 24 h **(H)** or 48 h (**A**–**G**), **(I)**. **(A)** The data shown are representative histograms of activated or control OT-I T cells’ forward scatter area (FSC-A) or expression of the indicated cell surface or intracellular markers as assessed by flow cytometry analysis. The values in histograms are geometric mean fluorescence intensities (MFI). Paired biological replicate comparisons of activated control and 3-MP treated OT-I T cells from repeat flow cytometry experiments show the geometric MFI of FSC-A **(B)**, CD69 **(C)**, CD71 **(D)**, PD-1 **(E)**, Tbet **(F)**, and CD25 **(I)** (*n* = 3-7). The proportions of live cells were determined by flow cytometry (*n* = 5) **(G)**. Paired biological replicate comparisons of levels of supernatant IL-2 were assessed by using ELISA (*n* = 3) **(H)**. In all cases, the dots joined by lines represent paired samples from an independent experiment. NS, not significant as determined by ratio paired *T*-test.

**Figure 3:**
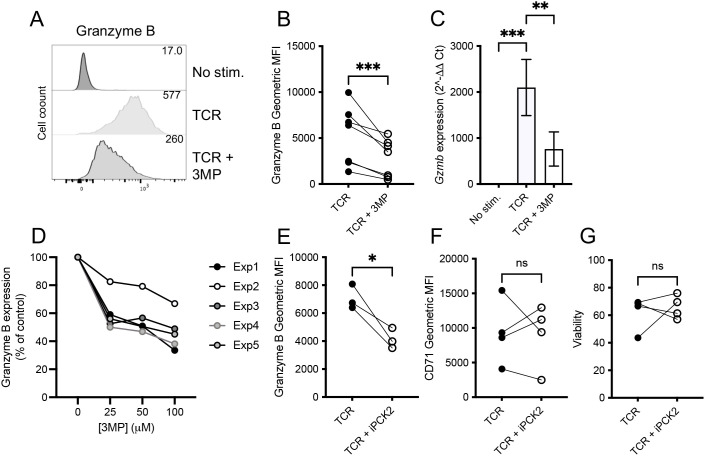
The PEPCK inhibitors reduce the TCR-induced upregulation of granzyme B expression. OT-I TCR transgenic T cells were stimulated with cognate peptide SIINFEKL (TCR) ± 3-MP (25–100 μM, as indicated) or iPCK2 (5 μM) for 48 h. **(A)** Representative histograms of intracellular granzyme **(B)** The values in histograms are geometric mean fluorescence intensities. **(B)** The paired biological replicate comparisons of activated control and 3-MP treated OT-I T cells from repeat flow cytometry experiments show the geometric MFI of granzyme B (*n* = 7). **(C)** 3-MP limits TCR-induced *Gzmb* transcription as determined by qRT-PCR. The values represent means ± SD from biological replicates (*n* = 4). **(D)** Dose-dependent inhibition of granzyme B expression by 3-MP. Each line represents data from an independent experiment with values normalized to no inhibitor control samples. iPCK2 inhibits TCR-induced impede granzyme B expression **(E)** but not CD71 **(F)** or cell viability **(G)** (*n* = 4 to 5). In all cases, the dots joined by lines represent paired samples from an independent experiment **(B)**, (**E**–**G**). NS, not significant. *p < 0.05, **p < 0.01, ***p < 0.001 as determined by ratio paired *T*-test or one-way ANOVA **(C)**.

To assess the role of PEPCKs in T cell activation, we used the well-characterized inhibitor 3-mercaptopicolinic acid (3-MP) (30, 31). 3-MP is a competitive inhibitor of PEP/OAA binding and binds a second allosteric site in the PEPCK structure (30). The CD8^+^ OT-I T cells were stimulated *in vitro* with cognate SIINFEKL peptide ± 3-MP for 48 h, and T cell activation was assessed. The flow cytometry analysis demonstrated that 3-MP did not impact on T cell growth, as assessed by FSC-A analysis, nor upon the TCR-induced upregulation of activation marker CD69, transferrin receptor CD71, exhaustion-associated immune checkpoint receptor PD-1, or transcription factor Tbet (**Figures 2A–F**; **Supplementary Figures 2B–D**). Furthermore, 3-MP did not affect the viability of activated OT-I T cells (**Figure 2G**), TCR-induced IL-2 production (**Figure 2H**), or IL-2Ra/CD25 expression (**Figure 2I**). By contrast, 3-MP limited the TCR-induced upregulation of cytolytic effector granzyme B protein (**Figures 3A, B**) and mRNA (**Figure 3C**) expression in a dose-dependent manner (**Figure 3D**). Importantly, a second structurally distinct PEPCK inhibitor, iPCK2 (28), also selectively inhibited TCR-induced granzyme B but not CD71 expression or cell viability in OT-I T cells (**Figures 3E–G**).

**Figure 4:**
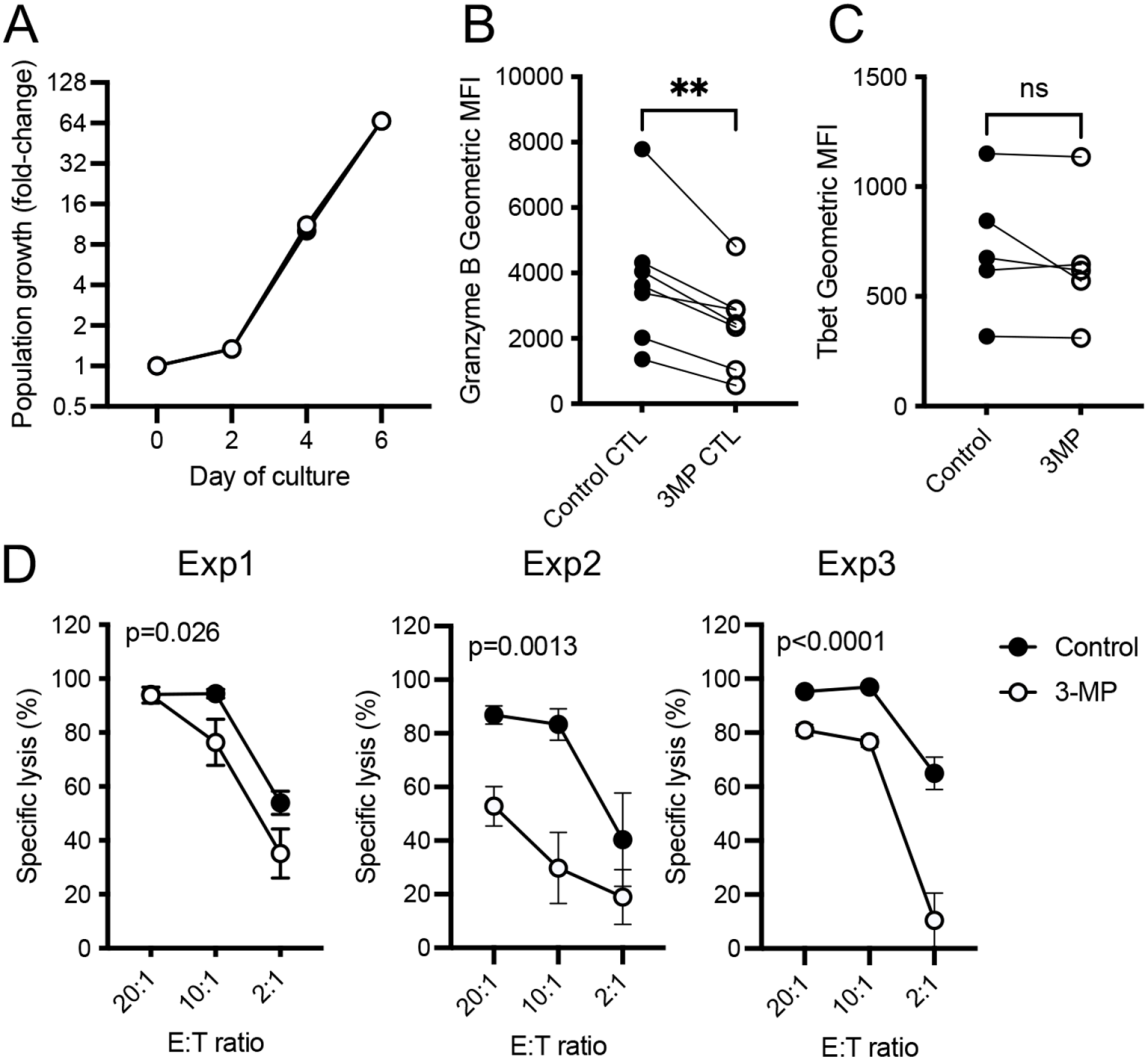
PEPCK inhibition reduces *in vitro* CD8^+^ T cell cytolytic capacity. **(A)** Effector CTLs were generated by 2 days of stimulation with SIINFEKL peptide, followed by 4 days of culture with high-dose IL-2 in the presence or absence of 100 μM 3-MP. The population expansion of activated OT-I T cells was determined by viable cell counting and is expressed as fold change. The values represent mean values from *n* = 3 biological replicates. Granzyme B **(B)** and Tbet **(C)** expression levels in day 6 CTLs were determined by intracellular staining and flow cytometry. The values represent geometric MFI from paired biological replicate samples from independent experiments (*n* = 5–7). **p < 0.01; ns, not significant as determined by paired Student’s *t*-test. **(D)** Specific lysis of ID8-OVA target cells by control or 3-MP OT-I CTLs at the stated effector/target ratios (E:T) in three independent experiments (Exp1– Exp3). The error bars represent SD from *n* = 3 replicate samples. The statistical analysis was carried out by using two-way ANOVA.

To determine if this reduced level of granzyme B expression impeded cytotoxic activity, effector OT-I CTLs were generated in the presence or absence of 3-MP by stimulating the cells for 2 days with SIINFEKL peptide followed by 4 days of differentiation using high-dose IL-2 as per our previously described protocol (32). Surprisingly, 3-MP did not impede the population expansion of OT-I CTLs over the course of the 6-day differentiation protocol (**Figure 4A**), while the analysis of antigen-induced T cell proliferation, by assessing the dilution of cell trace violet, similarly demonstrated a negligible effect of 3-MP (**Supplementary Figure 2D**). However, as was the case following 48 h of activation, the day 6 CTLs generated in the presence of 3-MP (hereafter termed “3-MP CTLs”) had reduced levels of granzyme B, but not Tbet, compared with control CTLs (**Figures 4B, C**). Furthermore, 3-MP OT-I CTLs had impaired capacity to kill OVA-expressing ID8 tumor cell targets *in vitro*, as shown by a shift in the titration curve (**Figure 4D**).

Next, we assessed the capacity of 3-MP CTLs to clear tumors *in vivo* using an adoptive T cell transfer (ACT) model. EL4-OVA lymphoma cells were injected into the flanks of C57BL/6 mice and allowed to establish tumors for 5 days, following which the mice received intravenous ACT using high numbers (5 × 10^6^) of either control or 3-MP OT-I CTLs. The data indicated that both control and 3-MP CTLs were competent to control EL4-OVA tumor growth in all of the mice assessed (**Supplementary Figure 3**). Taken together, these data indicate that 3-MP has a selective inhibitory effect on CD8^+^ T cell activation, reducing granzyme B expression and limiting *in vitro* CTL activity, but not population expansion. However, at high effector/target ratios or following ACT using high numbers of CTLs, 3-MP CTLs retain the capacity to kill cancer cells both *in vitro* and *in vivo*.

### PEPCK inhibition limits T cell inflammatory cytokine production

**Figure 5:**
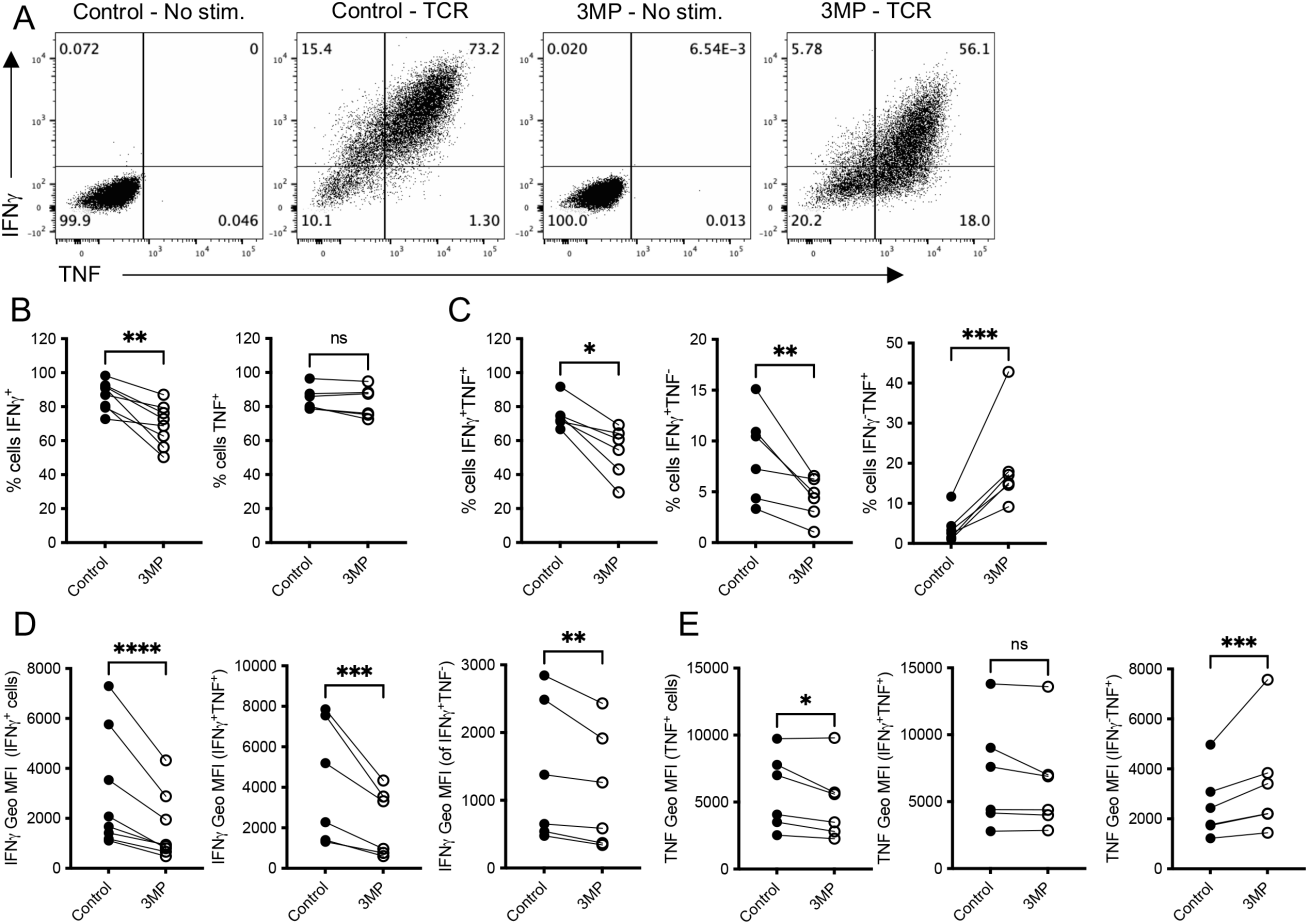
PEPCK inhibition limits CD8^+^ T cell inflammatory cytokine production. (**A**–**E**) Effector CTLs were generated by stimulation with SIINFEKL (2 days) and differentiation in IL-2 (4 days) in the presence or absence of 100 μM 3MP. The resultant CTLs were re-stimulated with SIINFEKL (TCR) and levels of IFNγ and TNF production assessed by FACS following gating on live cells. **(A)** Representative dotplots of IFNγ and TNF expression. **(B)** Proportions of total IFNγ^+^ and TNF^+^ cells following the re-stimulation of OT-I CTLs. **(C)** Proportions of IFNγ^+^TNF^+^, IFNγ^+^TNF^-^, and IFNγ^+^TNF^+^ following the re-stimulation of OT-I CTLs. The graphs show the geometric mean fluorescence intensity of IFNγ **(D)** and TNF **(E)** cytokine staining in gated total-positive, double-positive (IFNγ^+^TNF^+^) or single-positive (IFNγ^+^TNF^-^/IFNγ^+^TNF^+^) cells from paired biological replicate samples taken from independent experiments (*n* = 6). NS, not significant; *p < 0.05, **p < 0.01, *** *p* <0.001, ****p < 0.0001 as determined by paired Student’s *t-*test.

We set out to determine whether PEPCK inhibition impacted upon the acquisition of other T cell effector functions. To this end, day 6 OT-I CTLs generated in the presence or absence of 3-MP were re-stimulated with SIINFEKL peptide and effector cytokine production assessed by intracellular staining and flow cytometry. The proportions of 3-MP CTLs competent to produce IFNγ, but not TNF, were reduced (**Figures 5A, B**). A further analysis demonstrated that the decreased proportions of 3-MP CTLs were IFNγ+TNF^+^ “double-producers” and IFNγ+TNF^-^ “single-producers”, whereas the higher proportions were IFNγ−TNF^+^ single-producers, compared with control CTLs (**Figure 5C**). An analysis of the levels of IFNγ and TNF produced on a per cell basis, as assessed by geometric mean fluorescence intensity of staining, demonstrated that IFNγ+TNF^+^ double-producers typically produced higher levels of cytokines than single-producers for both control and 3-MP CTLs (**Figures 5D, E**). However, the levels of per-cell IFNγ were reduced by ~50% in both double-producer IFNγ+TNF^+^ and single-producer IFNγ+TNF^-^ 3-MP CTLs compared with the corresponding control CTL populations (**Figure 5D**). By contrast, the levels of per-cell TNF were unaffected in double-producer IFNγ+TNF^+^ and slightly elevated in single-producer IFNγ−TNF^+^ 3-MP CTLs compared with the corresponding control CTL populations (**Figure 5E**). Despite this, the average TNF geometric mean fluorescence in all gated TNF^+^ cells was mildly but statistically significantly reduced in 3-MP treated cultures compared with the controls (**Figure 5E**), reflecting the reduced proportions of the potent IFNγ+TNF^+^ double-producers present in these conditions. Further experiments demonstrated that CTLs grown in the presence of an alternative inhibitor, iPCK2, also had a reduced capacity to produce inflammatory cytokines, particularly IFNγ, upon antigenic restimulation compared with control CTLs (**Supplementary Figure 4**).

**Figure 6:**
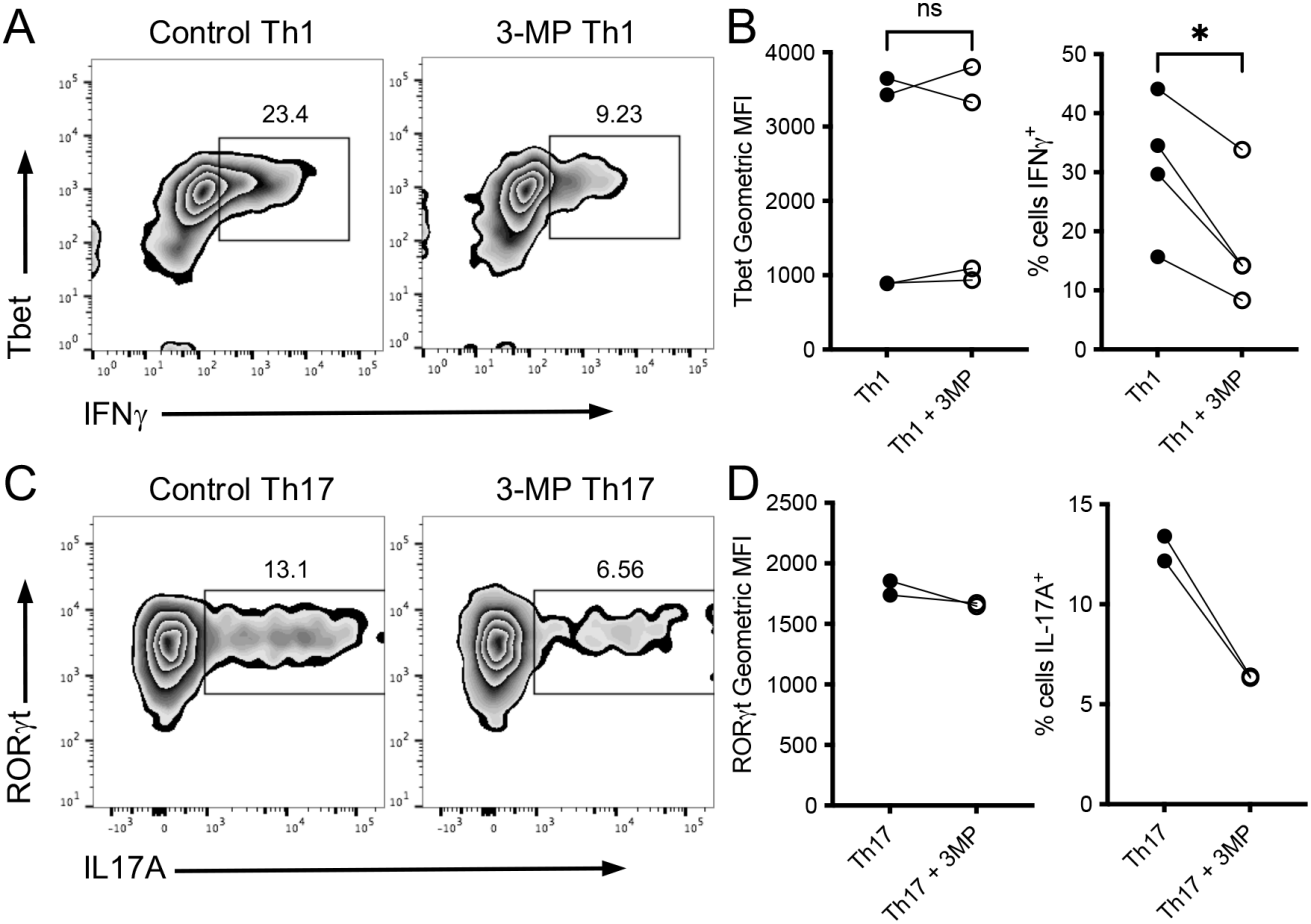
PEPCK inhibition limits CD4^+^ T cell inflammatory cytokine production. The purified C57BL/6 CD4^+^ T cells were stimulated under Th1-polarizing conditions for 3 days **(A, B)** or Th17-polarizing conditions for 6 days **(C, D)** in the presence or absence of 100 μM 3-MP and levels of lineage-specific transcription factor and cytokines assessed by flow cytometry. The dot plots show the representative analyses of intracellular staining for Tbet and IFNγ **(A)** and RORγt and IL-17A **(C)**. In all of the graphs, the circles represent values from paired biological replicate samples from independent experiments (*n* = 2–4). NS, not significant; *p < 0.05 as determined by paired Student’s *t*-test.

It was of interest to determine whether 3-MP had similar effects on CD4^+^ T cell cytokine production. Polyclonal C57BL/6 lymph node CD4^+^ T cells were activated with CD3 and CD28 antibodies in the presence of IL-12 for 3 days, and the levels of the canonical Th1 cytokine IFNγ and transcription factor Tbet were assessed by using flow cytometry. As with the results determined for CD8^+^ T cells, 3-MP did not impact upon the upregulation of Tbet expression in CD4^+^ T cells but impeded IFNγ production (**Figures 6A, B**). In parallel experiments, the impact of 3-MP on Th17 cell differentiation was assessed. CD4^+^ T cells were activated under Th17-polarizing conditions for 6 days, and the levels of key Th17-associated transcription factor RORγt and IL-17A were assessed by using flow cytometry. Similar to the results for Th1 differentiation, the levels of RORγt were comparable in the control and 3-MP Th17 cells, whereas the proportion of IL-17A^+^ cells was reduced by ~50% by 3-MP (**Figures 6C, D**). These results together demonstrate that PEPCK inhibition does not impede the upregulation of lineage-defining transcription factors such as Tbet or RORγt during T cell differentiation but instead reduces the capacity of effector CD8^+^ and CD4^+^ T cells to produce inflammatory cytokines.

### Metabolic effects underpin the inhibitory effects of 3-MP

**Figure 7:**
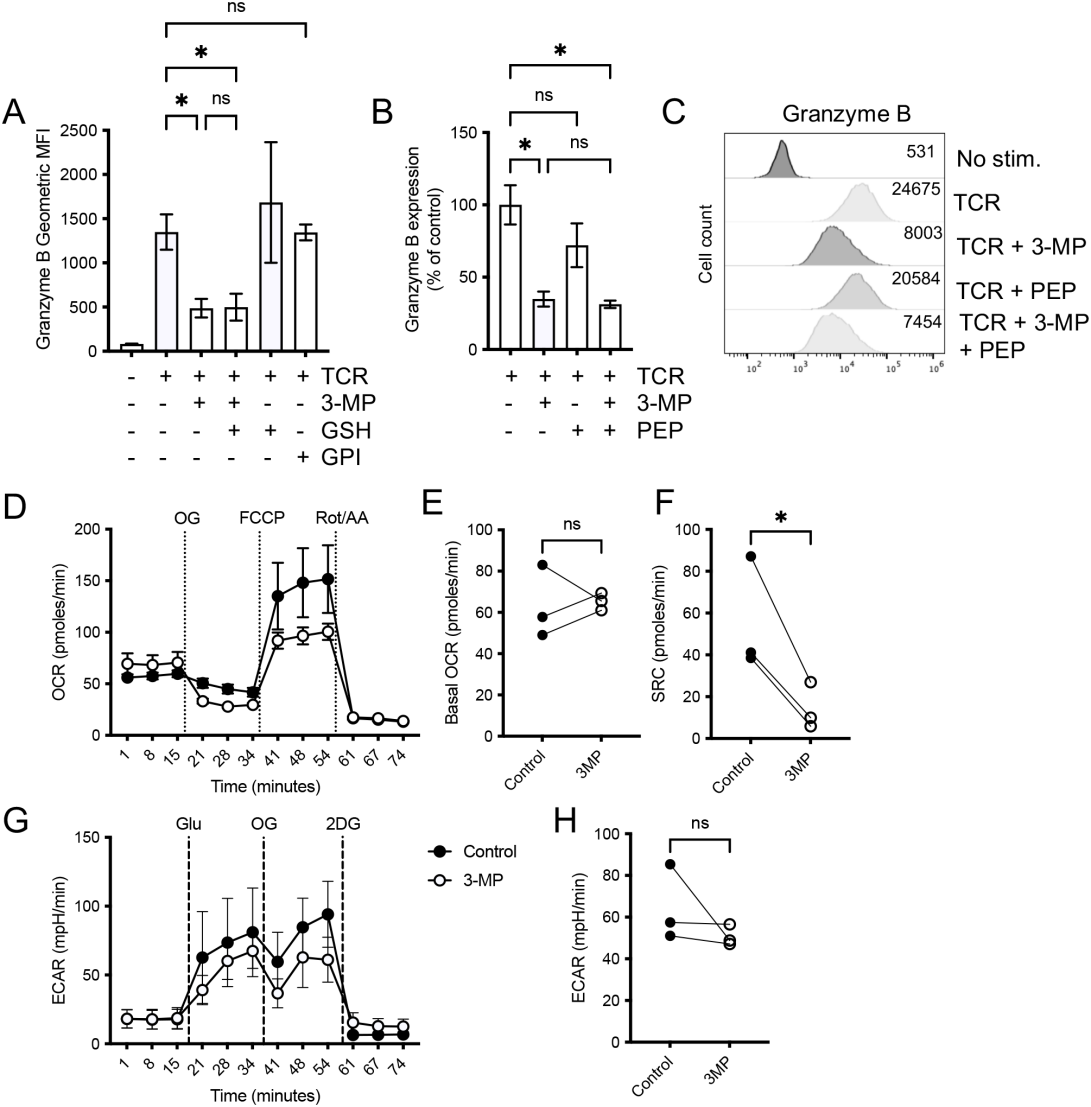
3-MP impacts upon mitochondrial respiration. The OT-I T cells were activated with SIINFEKL peptide (TCR) in the presence or absence of 100 μM 3-MP, 1 μM GSH, and 50 μM glycogen phosphorylase inhibitor (GPI) **(A)** or 3-MP and 1 mM PEP **(B, C)** for 48 h and granzyme B assessed by intracellular staining and flow cytometry. For (**A**, **B**), the bars represent mean values from biological replicate values (*n* = 3 to 4) ± SD. **(C)** Representative histograms showing the levels of intracellular granzyme B under the stated activation conditions. **(D)** The OT-I T cells were activated for 24 h ± 3-MP, and then the analysis of oxygen consumption rates (OCR) was performed using the Seahorse Mitostress test. The values represent means, and the error bars represent the SEM of technical replicates (*n* = 5) from one of three repeat experiments. The mean basal OCR **(E)** and spare respiratory capacity (SRC) **(F)** values were calculated from biological replicate experiments (*n* = 3). **(G)** The analysis of extracellular acidification rates (ECAR) was performed using the glycolysis stress test. The values represent means, and the error bars represent the SEM of technical replicates (*n* = 5) from one of three repeat experiments. **(H)** The glycolysis (glucose-induced ECAR – basal ECAR) values were calculated from replicate experiments (*n* = 3). NS, not significant; *p < 0.05 as assessed by one-way ANOVA, with Holm–Sidak correction for multiple comparisons **(A, B)** or paired Student’s *t*-test (**E**, **F**, **H**).

We sought to define the mechanisms underpinning the effects of 3-MP on inflammatory T cell effector responses. Previous reports indicated that the effects of PEPCK-C inhibition on memory T cells could be reversed by the addition of GSH to T cell cultures and were phenocopied by glycogen phosphorylase inhibitors (GPI) (22). Therefore, we assessed the impact of GSH supplementation and GPI on TCR-induced OT-I T cell granzyme B expression in the presence or absence of 3-MP. The addition of GSH did not reverse the 3-MP-mediated inhibition, while GPI had no effect on TCR-induced granzyme B expression (**Figure 7A**). These data indicate that the effects of 3-MP reported here are mechanistically distinct from the effects of *Pck1* deletion in previous studies. A further possibility was that the 3-MP treatment limited T cell activation by impacting on the cellular abundance of PEP. However, the addition of PEP to the T cell culture media did not alleviate the effects of 3-MP inhibition (**Figures 7B, C**).

Metabolic analyses using the Seahorse analyzer Mitostress test indicated that OT-I CD8^+^ T cells activated for 24 h in the presence of 3-MP had a comparable basal oxygen consumption rate (OCR) (**Figures 7D, E**), but reduced spare respiratory capacity (SRC) (**Figure 7F**), relative to the control cells. A further analysis, using the glycolysis stress test and assessment of extracellular acidification rate (ECAR), indicated that the 3-MP-treated cells displayed a trend for lower glycolysis; however, this did not reach statistical significance (**Figures 7G, H**). These data suggest that PEPCKs in activated T cells have subtle effects in mitochondrial metabolism, while 3-MP treatment impedes these functions.

## Discussion

In this paper, our initial analyses focused on assessing which PEPCK isoforms were expressed during T cell activation. The analysis of mouse T cell proteomic and transcriptomic datasets indicated that PEPCK-M is expressed in effector T cell populations. Neither PEPCK-C protein nor its transcript *Pck1* was detected in resting or effector populations, consistent with previously published data indicating that PEPCK-C is expressed selectively in memory T cells (22). Further experiments determined that pharmacological PEPCK inhibition impedes T cell cytotoxic function and inflammatory cytokine production. These data implicate PEPCKs as key metabolic regulators of T cell effector function and as potential targets to modulate T cell metabolism and function.

PEPCK-C is a rate-limiting enzyme in hepatic gluconeogenesis with a high protein expression detected in the kidney, gut, and liver (33). A previous study indicated that this isoform was upregulated in memory T cells but was low/undetectable in naïve and effector T cells (22). Here PEPCK-C protein and *Pck1* mRNA were not detected in naïve and effector mouse T cell subsets in publicly available proteomic and RNA-Seq datasets, whereas PEPCK-M/*Pck2* was widely expressed. These data suggest that PEPCK-C has an important role in T cell memory, whereas PEPCK-M may have a role in T cell effector functions. Consistent with this, *Pck1*-haploinsufficiency does not impair effector T cell responses during bacterial infections but limits the generation of memory T cell populations (22). Nonetheless, the current study, using inhibitors that affect both PEPCK isoforms, does not specifically preclude an involvement of either isoform in T cell effector responses.

3-MP was established as an inhibitor of PEPCK in the 1970s (31, 34) and, more recently, was shown to act as a competitive inhibitor of PEP/OAA binding and to bind a second allosteric site in the PEPCK structure (30). Throughout the current study, we used concentrations of 3-MP and iPCK2 previously shown to have a high specificity for PEPCK (28, 35). Nonetheless, we are mindful that there is always the possibility of unexpected, off-target effects in any pharmacological study and that, in future studies, genetic approaches will be required. In the present work, we showed that 3-MP did not have a global inhibitory effect on T cell activation but instead reduced the maximal expression of granzyme B in CD8^+^ T cells and inflammatory cytokine production by both CD4^+^ and CD8^+^ T cells. A second structurally distinct inhibitor, iPCK2 (28), also reduced granzyme B expression and cytokine production, strengthening the evidence for a role for PEPCKs in these processes. CTLs generated in the presence of 3-MP were less cytotoxic than the control counterparts on a cell/cell basis but were able to kill target cells *in vitro* and control tumor growth *in vivo* if supplied in sufficient numbers. Furthermore, 3-MP did not impair Th1 and Th17 differentiation *per se*, as the levels of lineage-defining transcription factors Tbet and RORγt were not impaired, but rather reduced the capacity of T cells to produce high levels of cytokines. Previous studies linked PEPCK-C expression in memory T cells to the maintenance of GSH levels and redox balance via the regulation of glycogen synthesis and metabolism (22). By contrast, neither GSH nor glycogen phosphorylase inhibition affected granzyme B expression in control or 3-MP-treated T cells, suggesting a different mechanism being key to the results of the present study. PEP supplementation did not rescue the effects of 3-MP on TCR-induced granzyme B expression, suggesting that 3-MP does not inhibit T cell activation simply via limiting the PEP levels. Given that 3-MP did not appear to impede the glycolytic flux, it is possible indeed that enolase-dependent PEP production compensates for direct effects on PEP levels resulting from PEPCK inhibition. It is worth noting that cytotoxic effector proteins such as granzyme B are among the most abundant proteins expressed by CTLs (36). Selective reductions in granzyme B and cytokine expression following 3-MP treatment implies that PEPCK function is important when the demand for gene transcription and protein synthesis is high. Our metabolic analyses determined that 3-MP-treated CD8^+^ T cells had reduced maximal, stressed mitochondrial respiration rates but had similar levels of glycolytic flux. Similarly, previous studies determined that the downregulation of *PCK2* impairs OXPHOS in cancer cells (19). However, the lack of effect of the inhibitors on T cell population expansion suggests that there are likely more subtle effects of PEPCK inhibition rather than a simple reduced capacity for energetic metabolism.

Our working hypothesis is that the inhibition of PEPCK-M underlies the effects of 3-MP and iPCK2 on T cell activation. Nonetheless, we cannot formally rule out a role for either isoform at present. Recent studies have shown that *Pck2^-/-^* mice are viable and fertile (37) and that PEPCK-M plays a role in the maintenance of a mitochondrial PEP cycle, alongside pyruvate carboxylase and pyruvate kinase, that is important to regulate pancreatic β cell insulin secretion (37, 38). A role for PEPCK-M in regulating LPS-driven Kupffer cell inflammatory responses has been suggested (39). Future work will require an in-depth analysis of immune phenotypes and anti-tumor immune responses of T cell-conditional *Pck1^-/-^* and *Pck2^-/-^* mouse strains to address the question of the precise role of PEPCK-C and PEPCK-M in T cell effector responses. Furthermore, metabolomic analyses of control and *Pck1^-/-^* and *Pck2^-/-^* T cells will reveal the precise consequences of PEPCK function during effector T cell responses.

While the 3-MP-treated OT-I T cells had impaired effector functions *in vitro*, our adoptive T cell transfer experiments showed that they were still competent to clear EL4-OVA tumors. It is possible that the *in vivo* defects of 3-MP CTLs might be revealed by using lower numbers of CTLs in ACT experiments or by treating tumor-bearing mice directly with PEPCK inhibitors. In this regard, several recent studies have reported the differing effects of 3-MP treatment on *in vivo* T cell responses to tumors. Ma and colleagues reported that the 3-MP treatment resulted in the accelerated growth of B16-OVA melanoma, while *Pck1* heterozygous OT-I T cells were inferior to control cells in controlling tumor growth following ACT (22). The adoptive transfer of PEPCK-C-overexpressing tumor-reactive T cells has been shown to result in more effective tumor control than control T cell ACT in several studies (20, 22). By contrast, a recent study reported that 3-MP treatment decreased Treg proliferation and increased the proportions of IFNγ-producing CD8^+^ T cells within B16 tumors, without impacting overtly on overall tumor growth (40). The interpretation of these studies is complicated as 3-MP will have direct and indirect effects on many different cell populations *in vivo*, and the overall outcome on tumor growth and anti-tumor immunity will be highly context-dependent. Nonetheless, these previous studies and the work presented here add to a growing body of evidence that implicates PEPCK isoforms as key regulators of T cell metabolism and activation that can be targeted to influence T cell responses.

## Data Availability

The raw data supporting the conclusions of this article will be made available by the authors, without undue reservation.
