# Supplementary material for: Phospho-enol pyruvate carboxykinase inhibition limits effector function in inflammatory T cells
Source: Front Immunol. 2026 Apr 13;17:1706167. doi: 10.3389/fimmu.2026.1706167 (PMC13111197; doi:10.3389/fimmu.2026.1706167)
Supplement: Supplementary file 1 [file SupplementaryFile1.docx]

**Phospho-enol pyruvate carboxykinase inhibition limits effector function in inflammatory T cells**

Rebecca J. Brownlie^1^*, Helen Carrasco Hope^1,2^*, David Wright^3^, Graham P. Cook^1^, José C. Perales^4^, Robert J. Salmond^1^

1. Leeds Institute of Medical Research at St. James’s, University of Leeds, Wellcome Trust Brenner Building, St. James’s University Hospital, Leeds, United Kingdom

2. Faculty of Science and Medicine, Université de Fribourg, Fribourg, Switzerland

3. Institute of Immunology and Infection Research, University of Edinburgh, Ashworth Laboratories, Edinburgh, United Kingdom

4. Department of Physiological Sciences, School of Medicine, University of Barcelona, L’Hospitalet del Llobregat, Spain

* Contributed equally

Correspondence: Robert J. Salmond, [r.j.salmond@leeds.ac.uk](mailto:r.j.salmond@leeds.ac.uk)


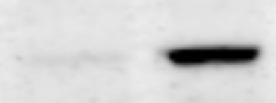

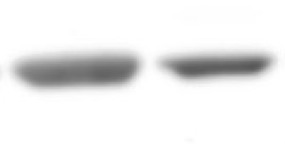

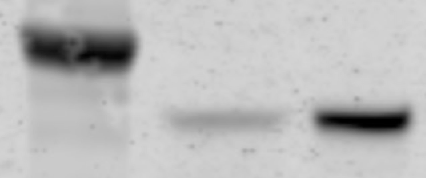

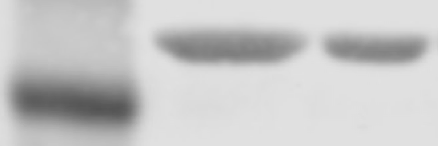


80kDa

30kDa

Naïve

Effector

Naïve

Effector

PEPCK-M

Actin

PEPCK-M

Actin

A

B

**Supplementary Figure 1.** Repeat experiments showing PEPCK-M expression in naïve and effector OT-I T cells (related to Figure 1D).

B

C

D

CTV

E

IL-7

TCR

TCR/3MP

Cell number


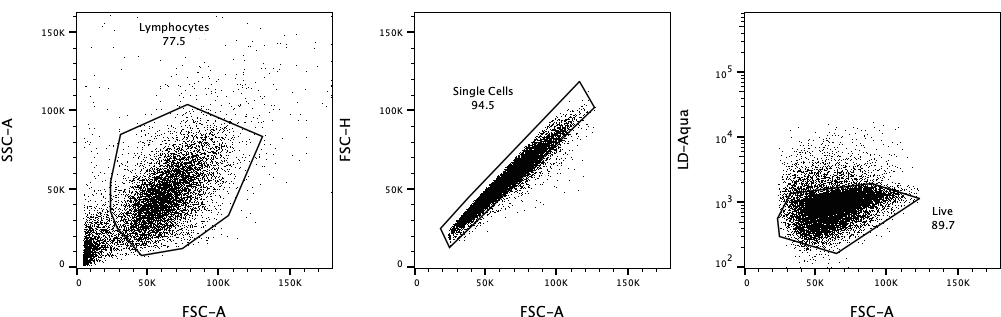


Lymphocyte gate

Singlet gate

Live-dead discrimination

A

**Supplementary Figure 2.** PEPCK inhibition does not impede TCR-induced upregulation of activation markers or proliferation. (**A**) Gating strategy to determine cell viability. (**B-D**) OT-I T cells were activated with SIINFEKL peptide ± 3MP at the stated concentrations for 48h. Comparisons of activated control and 3-MP treated OT-I T cells from repeat flow cytometry experiments show relative levels of expression of of CD71 (**B**), PD-1 (**C**) and Tbet (**D**). In all cases, values are normalised to the no inhibitor control in each experiment and error bars represent SD. N>4 biological replicates in all cases. NS = not significant, as determined by One-way ANOVA. (**E**) OT-I T cells were loaded with cell trace violet (CTV) and activated with SIINFEKL peptide ± 100μM 3MP for 72h. Histograms show representative results of CTV dilution from 1 of 2 repeated experiments.

**
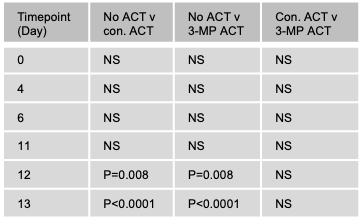
**

A

B

C

No ACT

Control OT-I ACT

3MP OT-I ACT

**Supplementary Figure 3.** CTLs generated in the presence of 3-MP can clear tumours *in vivo.* (**A**) Groups of mice (n=5/group) were challenged with s.c. EL4-OVA and following 5 days received no ACT, control OT-I or 3-MP OT-I CTL ACT (5x10^6^ cells/mouse). Data shown are mean tumour volume ± SEM as assessed by caliper measurements. (**B**) Statistical analysis of mean tumour volumes from part A, as determined by two-way ANOVA. (**C**) EL4-OVA tumour growth in individual mice. Data show tumour volumes as determined by caliper measurements, each line represents an individual mouse within each treatment group (n=5/group).


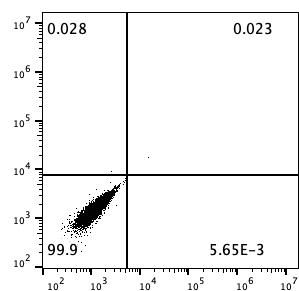

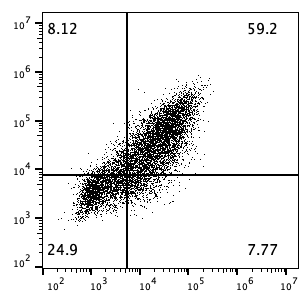

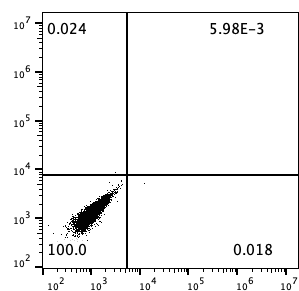

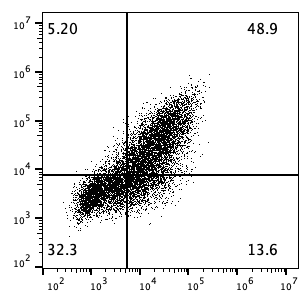


Control - No stim.

iPCK2 - No stim.

Control - TCR

iPCK2 - TCR

IFNγ

TNF

A

B

**Supplementary Figure 4. i**PCK2 inhibitor limits CD8^+^ T cell inflammatory cytokine production. Effector CTLs were generated by stimulation with SIINFEKL (2d) and differentiation in IL-2 (4d) in the presence or absence of 5μM iPCK2. Resultant CTLs were restimulated with SIINFEKL (TCR) and levels of IFNγ and TNF production assessed by FACS. (**A**) Representative dotplots of IFNγ and TNF expression. (**B**) Graphs show proportions of IFNγ^+^TNF^+^, IFNγ^+^ and TNF^+^ OT-I CTLs. Values are from paired biological replicate samples from independent experiments (n=3). * p<0.05, ** p<0.01, as determined by paired Student’s *t-*test.
